# Intensive Laboratory experiences to safely retain experiential learning in the transition to online learning

**DOI:** 10.1002/ece3.6886

**Published:** 2020-11-03

**Authors:** Marcus Lashley, Robert McCleery

**Affiliations:** ^1^ Wildlife Ecology and Conservation University of Florida Gainesville FL USA

**Keywords:** COVID‐19, field work, flipped classroom, online pedagogy, student‐focused

## Abstract

Field‐based course work has been foundational to Ecology and Evolutionary Biology curricula. However, opportunities for these experiences gradually have decreased over the past few decades and are being replaced with technology in the college learning environment. The coronavirus disease 2019 pandemic facilitated a rapid transition of all field‐based courses to online only delivery, which we argue has forced us to reconsider how to deliver course content to retain field experiences in a manner that is safe during the pandemic but robust to ever changing constraints in the college classroom. Here, we propose pairing an intensive laboratory experience with an otherwise online delivery. We discuss several advantages of intensive laboratory experiences that occur in the field over a short but intensive time period over that of the traditional low‐intensity weekly laboratory structure. In particular, intensive laboratory experiences are safer during the pandemic because they allow the group to be tested and isolated, allow more flexibility for students with competing interests for their time, and also enhance student interpersonal skills while still providing strong reinforcement of the skills typically honed through experiential learning. We present case studies for how we intend to apply our proposed model to two courses that heavily rely on field‐based experiential learning to facilitate adoption.

## Graphical abstract

We propose an intensive lab experience can safely retain experiential learning in the field during the pandemic and provide a number of advantages to the traditional lab model. Here we provide two examples of how we plan to apply our proposed model to field based courses.

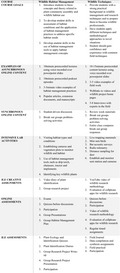

## 1 INTRODUCTION

Field‐based course work has been a foundational part of Ecology and Evolutionary Biology (EEB) curricula because of the undisputed value of learning by doing. We realize that it is difficult to appreciate the evolutionary significance of the enlarged anal scent gland in skunks (Mephitidae) without having smelled it or the effectiveness of epizoochory as a dispersal strategy until removing beggar's lice (*Desmodium* sp.) from one's pant leg. It is important for students to experience the processes and concepts they are learning about nature in nature. Experiential learning provides students ample time and space to engage in this process of learning through the senses. This hands‐on approach allows students to contextualize information by experiencing nature and to build knowledge by assimilating new experiences through self‐reflection (Kolb & Kolb, 2005; Kolb, 2014; Kolb et al., 2001; Lewis & Williams, 1994). In comparison with knowledge transfer‐based strategies, experiential learning is superior in fostering knowledge retention and application (Kolb & Kolb, 2006; Springer et al., 1999). This is especially true in EEB where many students choose the disciplines because of interest and excitement they garnered from previous experiences with nature. Field‐based experiential learning strategies also decrease the knowledge gap between students across disciplines (Prestholdt & Fletcher, 2018) and improve the achievement and graduation rates for underrepresented groups (Beltran et al., 2020). Unfortunately, changing societal constraints have gradually reduced the number of field‐based learning opportunities over the past decades (Fleischner et al., 2017; Hafner, 2007; Schmidly, 2005).

Over the last several decades, we have seen a marked decline in field‐based experience for collegiate undergraduates in EEB (Fleischner et al., 2017). Consequently, many EEB students have not been adequately trained and are unable to conduct the field‐based research (Noss, 1996) essential for advancing ecology and solving our planet's conservation problems (Barrows et al., 2016; McGlynn, 2008). This decline has been precipitated by the growing constraints on university administrations, students, and instructors. For university administrations, field‐based experiences can be expensive, must be limited to designated class times, and bring potential liabilities for student safety (i.e., field activities and travel) and animal ethics concerns. These limitations have curbed traditional field‐based activities like the capture of animals at remote field sites, to the point that they are quickly becoming an anomaly (Fleischner et al., 2017).

As a result of many constraints on field activities, many ecological‐based classes are now conducted locally (e.g., on or around campus) during regularly scheduled class laboratories (Fleischner et al., 2017; McCleery et al., 2005). While it is possible to create meaningful field experience under these conditions (McCleery, 2015; McCleery et al., 2005), the growing demands on students time (course work, family, jobs, personal, internships, student clubs) has limited some students ability to attend courses that extend over multiple periods (Lei, 2010). Regular field‐based laboratory experiences also provide few alternatives for missed assignments and lack the flexibility students prefer in their schedules (McCleery, 2015). In addition, generational shifts in student background and comfort with nature have left an increasing number of urban and suburban students daunted, disinterested, or unable to understand the relevance in field‐based experiences (Cotton, 2009; Fleischner et al., 2017).

From a faculty perspective, developing and conducting field‐based classes require more time, effort, and creativity than lecture or discussion‐based courses (Lei, 2010; McCleery, 2015; McCleery et al., 2005). In addition to addressing safety and ethical issues, instructors need to plan for and manage logistics, finances, equipment and permits for various laboratories and experiences (Fleischner et al., 2017). These additional burdens along with the growing demands for research output and student‐driven mentoring can discourage even dedicated faculty from starting or continuing field‐based experiences (Fleischner et al., 2017; Lei, 2010; McCleery et al., 2005).

Already facing a number of formidable hurdles to providing meaningful field experience to undergraduates, instructors were recently asked to rapidly transition to remote instruction due to the COVID‐19 pandemic (Crawford et al., 2020). The pandemic may have forced rapid changes that were seemingly inevitable. In a matter of weeks or even days, field‐based disciplines around the globe were forced to abruptly transition courses to an online form (Corlett et al., 2020). With few exceptions (i.e., backyard experience, digital field laboratories), field experiences were eliminated or greatly reduced. Now with the onset of the Fall 2020 semester, instructors have been asked to reimagine and design their courses in new formats that maximize online learning as well as safety of students and faculty. In essence, the pandemic has forced us to reconsider how we approach learning in EEB altogether (Lashley et al., 2020). Like perhaps no other event in the history of higher education, the pandemic has aligned the interest of educators around the globe on a single central question—how do we take learning to online delivery effectively (Lashley et al., 2020)? Most EEB educators would agree that losing the field experiences is not an option. We suggest that it is possible to maximize online learning, prioritize student safety, and reimagine the format of meaningful field experience in EEB. While there is no precedent or pedagogical foundation for developing field experience during a pandemic, we believe the pandemic has given us an opportunity to rethink student field experiences in a way that is robust to the situation fostered by the pandemic but also to the ever‐increasing constraints on the status quo.

## 2 INTENSIVE LABORATORY EXPERIENCES AS A NEW MODEL FOR EEB

The most common prepandemic model for EEB field courses was in‐person lectures paired with a quarter or semester long recurring outdoor laboratory or field trips. Given the aforementioned obstacles and the seeming insurmountable constraints of the pandemic, there is a need for viable alternatives that preserve or even enhance the field experience. To promote safety and maximize the benefits and field experiences, we recommend short intense (5–7 days) outdoor laboratory experiences (hereafter, intensive laboratory experiences; Bogner, 1998; Lisowski & Disinger, 1991). We believe that this approach has a number of critical advantages over weekly laboratories or regular field trips. First and foremost, short intensive field experiences at remote sites may be the best option for keeping students and faculty safe from exposure to COVID‐19. These intensive laboratory experiences would allow us to create a safe learning environment by testing and then isolating all participants (students and instructors) at a remote site. Coupled with face masks and sanitary practices, a tested and isolated group has greatly reduced risk of transmission (Cooper et al., 2020; Feng et al., 2020). Intensive field experiences also hold several practical and logistical advantages over regular laboratories and field trips. By integrating this one block of time (e.g., spring break, May‐mester, intersession) into student's schedules, it will be easier for students to make arrangements for jobs, family, and other commitments that might limit regular participation (Lei, 2010). Additionally, by moving to a remote location without the constraints of class periods, it eliminates wasted travel time (Lei, 2010) and allows for the timing of instruction to be flexible to capture critical events or time periods such as animal activity peaks. Additionally, working in remote natural settings makes learning more enjoyable while enhancing critical thinking, problem‐solving, and self‐confidence (Lei, 2010).

We also see several important advantages of short intensive immersive experience over laboratories and field trips. For example, working and living together, even over a short period of time enhances student's participation, leadership, independence, and confidence (Boyle et al., 2007; Fleischner et al., 2017). These experiences also foster interpersonal skills while helping to create meaningful relationships with faculty, professionals (e.g., nonfaculty instructors) and each other (Cuseo, 2018; Fleischner et al., 2017; Lei, 2010). These social bounds are increasingly important as online education has left many students feeling isolated and anxious (Cao et al., 2020). Finally, short intense immersion experience of 5–7 days enhances student knowledge and long‐term retention of ecological concepts (Lisowski & Disinger, 1991). Thus, we contend that designing online courses with intensive laboratory experiences could overcome the health concerns we currently face during the pandemic but also has the potential to improve the practicality and effectiveness of the field experiences over that of the traditional recurring weekly laboratory model.

## 3 REMODELING FIELD‐BASED CLASSES FOR ONLINE DELIVERY

Some EEB courses are inherently field intensive, where the knowledge and skills gained from instruction are heavily influenced or dependent on student engagement in the field. Here, we describe several techniques that can maximize the effectiveness of the online portion of the course and present two models of how we intend to adapt two inherently field‐dependent courses to an online format without losing the experiential learning aspects of the course by adding an intensive laboratory experience. Our intent was not only to design each course such that they accommodate obstacles posed by the pandemic but also to be robust to the steadily changing nature of the college classroom.

## 4 ONLINE DELIVERY FORMAT

This generation of university‐enrolled students is the first that has always had computer‐based technologies and the Internet while most of the instructors have not for the entirety of their life or even career. This divide between generations can be frustrating for students and the instructors because students expect to use technology in the classroom (McCleery et al., 2005; Millenbah et al., 2011; Pardue & Morgan, 2008) and often have little patience for traditional lecture‐based classes (Roehl et al., 2013). The abilities of the students have not changed, but how they learn has shifted substantially with technology (Prensky, 2010). Today, students have specific preferences for the design of the online classroom. They desire to express their own ideas, prefer working collaboratively on assignments, and respond positively to active learning by engaging in real‐world issues (Kraus & Sears, 2008; McGlynn, 2005; Millenbah et al., 2011; Roehling et al., 2010; Taylor & Keeter, 2010). Moreover, students now have competing interests for their time such as work, family, or social related activities (Hanson et al., 2010) and thus may appreciate pedagogical approaches that provide flexibility in scheduling (McCleery, 2015).

### 4.1 Asynchronous delivery

We propose an online delivery format that involves a flipped classroom pedagogical approach that has a synchronous, asynchronous, and intensive laboratory experience sections of the course. In the asynchronous portion of the course, short clips of media 5–15 min in length are prerecorded and made available in advance for students to watch/read/listen to when it suits their individual schedule but before the paired synchronous portion of the course ensues. We recommend different types of media to hold student attention, provide opportunities for different learning styles, and potentially increase engagement (Roehling et al., 2010). More standard short PowerPoint‐based video lectures (McCleery, 2015) should be paired with video clips, webpages, blogs, and handouts that demonstrate a practice, method, or concept, or explore a relevant topic. Additionally, instructors should consider delivering content through emerging platforms such as with podcasts or virtual environments. Podcasts are increasing in popularity as a method of information gathering among college‐aged students, have the flexibility to adapt content to the classroom, present the opportunity to interview experts on a topic, and are easy to record and produce (Strickland et al., 2020). Creating virtual experiences can also be an effective way to complement other forms of online delivery, especially to allow students to engage and explore content similar to what would be experienced in outdoor activities but the technical skills needed to develop this content may be a hindrance (Diwakar et al., 2016).

### 4.2 Synchronous delivery

Structuring the synchronous portion of the course as a discussion‐based session can enhance the learning environment by allowing it to be student‐driven. One successful approach to discussion is to use them to review and highlight key points from online content, and then separate students into small groups (i.e., breakout rooms) to apply the knowledge to a real‐world issue (McCleery, 2015). After a brief (i.e., 5 min) group discussion, the groups share their approaches and reflect on their strengths and shortcomings. This approach is conducive to online delivery as many of the commonly used platforms have a whiteboard function, chat windows, and breakout rooms allowing groups to interact in their groups independent of other groups but also have conversation and take notes as a whole. Furthermore, this online flipped classroom model has been successful in several disciplines within STEM (Herreid & Schiller, 2013).

## 5 INTENSIVE LABORATORY EXPERIENCE STRUCTURE

We propose an intensive laboratory experience format be paired with the online course to meet the health concerns imposed upon us by the pandemic but also to remain robust to constraints emerging by changes in society. Intensive laboratory experiences would be designed such that they occur over a 1‐ to 2‐week period and require students to stay on site after being tested. The intensive laboratory experience does not necessarily need to occur during the online course and has the flexibility to occur between semesters or during ecological or evolutionarily relevant phenology (e.g., migrations, flowering). A variety of approaches to information delivery could be adopted but in general, we suggest 1‐ to 3‐hr long field activities 2–3 times per day with 15 min—1‐hr long intermissions. Providing opportunities to work in groups, especially in research related projects, can be particularly effective to enhance experiential learning by strengthening student–student relationships and can even provide a tangible outcome to those students interested in following through with publication of their work (e.g., Westlake et al., 2020). This approach would allow adequate time to become immersed in the experience but still allow adequate time for needed breaks and reflection. It would allow students to experience diel time‐sensitive ecological phenomena such as dawn choruses by being conveniently located onsite. Moreover, a real advantage in addition to experiential learning to this approach is that students do not have the regular distractions academically from other classes and extramural activities allowing them to focus on the subject at hand. Evaluation of these types of experiences can be a challenge but having short low stakes testing opportunities such as regular quizzes and daily field practicals may be an effective measure of knowledge gain. Higher stakes instruments such as group presentations, research reports, and field practicums can be administered less frequently. Also, we encourage some credit to be given for creativity and student engagement.

## 6 TWO APPLICATIONS OF THE ONLINE COURSE DELIVERY PAIRED WITH AN INTENSIVE LABORATORY EXPERIENCE

Both Wildlife Habitat Management (WIS 4427C) and Wildlife Techniques (WIS 4945C) have been taught at the University of Florida as typical semester long courses with in‐person lectures or discussion and weekly field‐based laboratories. We have reformatted both of these courses to reflect our recommendations above. The courses will have an online synchronous and asynchronous delivery with a paired ILE at the end of the spring semester (Table 1). This strategy will allow students to use the online classroom to learn concepts, theories, and approaches to habitat management and wildlife techniques and then transition to the ILE during the intermission period between semesters. There is an added benefit for this timing for both classes. In Habitat Management, many assignments are dependent on plant phenology, and the timing the ILE during the early growing season will facilitate data collection in the group research project and allow students to generate their own plant video diaries. For Techniques, the end of the spring semesters can maximize wildlife activity (i.e., herpetofauna, breeding birds) with minimal potential for heat stress for any animal captured during technique applications.

**Table 1:** Examples of the flipped classroom online structure paired with an intensive laboratory experience for two field‐based courses in offered in the department of wildlife ecology and conservation at the University of Florida, USA

Course	Wildlife Habitat Management	Wildlife techniques
Course goals	Introduce students to basic concepts and theory related to plant community assembly and wildlife habitat use. To develop student skills in assessment of habitat conditions and the application of habitat management practices to address specific habitat needs. Develop student skills in the use of habitat management tools to apply habitat management concepts.	Provide students with a strong practical background in wildlife management and research techniques and to prepare them to become wildlife professionals. Have students apply different techniques and methodological approaches to novel situations. Student should gain confidence and experience with common field techniques.
Examples of asynchronous online content	10‐min prerecorded lectures using voice recorded over PowerPoint slides. 10‐min prerecorded podcast episodes. 3‐ to 5‐min video examples of habitat management practices. Popular articles, extension documents, and manuscripts.	10‐ to 15‐min prerecorded content lectures using voice recorded over PowerPoint slides 3–5 video examples of field work Weblinks to videos and wildlife project home pages 5–8 Interviews with experts in the field
Synchronous online content	Student‐driven discussion Breakout groups problem‐solving activities	Review week materials Breakout groups problem‐solving activities Entire class compares breakout group responses
Intensive laboratory activities	Visiting habitat types and conditions Establishing cameras and vegetation plots to monitor wildlife and habitat Use of habitat management tools such as drip torch, chainsaw, tractor, and implements Identifying key wildlife plants	Live trapping mammals Mist nets birds Bat acoustic surveys Radio telemetry Distance sampling for deer Establish and monitor sent station and cameras
Ile creative assignments	Video diary of plant identification Group research project	YouTube video of wildlife research methodology Evaluation of cell phone apps for wildlife research
Online assessments	Examinations Quizzes before discussions Participation Group Presentations Group Habitat Management Plan	Quizzes before discussions Participation Video of wildlife research methodology Evaluation of cell phone apps for wildlife research Regular timed assignments
Ile assessments	Plant Ecology and Identification Quizzes Plant Identification Diaries Group Research Project Write‐up Group Research Project Presentation Participation	Field Journal Data compilation and synthesis assignments Field practical Participation

For both classes, the structure of online content will include approximately 10‐min prerecorded lectures using voice recorded over PowerPoint slides (Table 1). Examples of lecture topics for Habitat Management will include principles of plant succession, wildlife habitat use, habitat assessment, use of prescribed fire, while Techniques lectures will include study design, data interpretation, passive detection methods, capture and handling wildlife, and radio telemetry. Both classes will also utilize short podcast episodes, videos, and various article types to mix up the delivery of content. The synchronous part of the courses will include weekly discussion with the classes divided into smaller groups of 15–30. These sessions will be used for applying course content, problem‐solving in breakout groups, and student‐driven discussions of current events and case studies. Both classes will find creative uses of digital resources for assignment and use a suite of assessment tools to accommodate different learning styles (see Table 1).

The ILE portion of these classes will be based at regional field stations and vary in length from 7 to 14 days. Each day will include structured activities, unstructured and discussion of relevant techniques and management strategies (Table 1). Structured and unstructured activities will rely heavily on providing safe hands‐on opportunities to engage in class materials. Additionally, unstructured time will provide students an opportunity to collect data for their group projects and assignments, and to complete data compilations, plant diaries, and field notebooks (see Table 1).

## 7 CONCLUDING REMARKS

While the COVID‐19 pandemic has forced immediate action from educators to move course delivery online, we contend this does not necessitate the loss of experiential learning in the field. We suggest intensive laboratory experiences can allow a safe, inclusive, and effective learning environment that is applicable during the pandemic and beyond. Intensive laboratory experiences over a short period of time (e.g., Felege et al., 2019) similar to approaches used in study abroad courses (e.g., Lutterman‐Aguilar & Gingerich, 2002) should be an effective model to retain the benefits of learning in the field that we value in Ecology and Evolutionary Biology programs without compromising safety for students and instructors.

## CONFLICT OF INTERESTS

None declared.

## AUTHOR CONTRIBUTION

**Marcus Lashley:** Conceptualization (equal); Investigation (equal); Writing‐original draft (equal); Writing‐review & editing (equal). **Robert McCleery:** Conceptualization (equal); Resources (equal); Writing‐original draft (equal); Writing‐review & editing (equal).

## Data Availability

Not applicable.
